# An Atlas of Variant Effects to understand the genome at nucleotide resolution

**DOI:** 10.1186/s13059-023-02986-x

**Published:** 2023-07-03

**Authors:** Douglas M. Fowler, David J. Adams, Anna L. Gloyn, William C. Hahn, Debora S. Marks, Lara A. Muffley, James T. Neal, Frederick P. Roth, Alan F. Rubin, Lea M. Starita, Matthew E. Hurles

**Affiliations:** 1 Department of Genome Sciences, University of Washington, Seattle, WA USA; 2 Department of Bioengineering, University of Washington, Seattle, WA USA; 3 Brotman Baty Institute for Precision Medicine, Seattle, WA USA; 4 Wellcome Sanger Institute, Hinxton, Cambridgeshire UK; 5 Department of Pediatrics & Department of Genetics, Division of Endocrinology, Stanford School of Medicine, Stanford University, Stanford, CA USA; 6 Department of Medical Oncology, Dana-Farber Cancer Institute, Boston, MA USA; 7 Broad Institute of MIT and Harvard, Cambridge, MA USA; 8 Department of Systems Biology, Harvard Medical School, Cambridge, USA; 9 Novo Nordisk Foundation Center for Genomic Mechanisms of Disease at Broad Institute, Cambridge, MA USA; 10 Donnelly Centre and Departments of Molecular Genetics and Computer Science, University of Toronto, Toronto, ON Canada; 11 Lunenfeld-Tanenbaum Research Institute, Sinai Health System, Toronto, ON Canada; 12 Bioinformatics Division, The Walter and Eliza Hall Institute of Medical Research, Parkville, VIC Australia; 13 Department of Medical Biology, University of Melbourne, Melbourne, VIC Australia

**Keywords:** Multiplexed assay of variant effect, Genome interpretation, Variant effect, Saturation mutagenesis, Functional genomics, Global alliance

## Abstract

Sequencing has revealed hundreds of millions of human genetic variants, and continued efforts will only add to this variant avalanche. Insufficient information exists to interpret the effects of most variants, limiting opportunities for precision medicine and comprehension of genome function. A solution lies in experimental assessment of the functional effect of variants, which can reveal their biological and clinical impact. However, variant effect assays have generally been undertaken reactively for individual variants only after and, in most cases long after, their first observation. Now, multiplexed assays of variant effect can characterise massive numbers of variants simultaneously, yielding variant effect maps that reveal the function of every possible single nucleotide change in a gene or regulatory element. Generating maps for every protein encoding gene and regulatory element in the human genome would create an ‘Atlas’ of variant effect maps and transform our understanding of genetics and usher in a new era of nucleotide-resolution functional knowledge of the genome. An Atlas would reveal the fundamental biology of the human genome, inform human evolution, empower the development and use of therapeutics and maximize the utility of genomics for diagnosing and treating disease. The Atlas of Variant Effects Alliance is an international collaborative group comprising hundreds of researchers, technologists and clinicians dedicated to realising an Atlas of Variant Effects to help deliver on the promise of genomics.

## Introduction

Two decades after sequencing the first human genome, millions of human exomes and genomes have been sequenced. Interpreting the effects of the hundreds of millions of variants thus discovered has become a central challenge for genomics. The genomes of the 8 billion people alive today collectively contain nearly all ~ 9 billion possible single nucleotide genetic variants compatible with life, as well as numerous insertions, deletions and other types of variants [1, 2]. Moreover, within the trillions of cells of each individual, every possible single nucleotide genetic variant will have arisen through somatic mutation. The functional impact of genetic variants has primarily been determined by asking if the variant co-occurs with a disease, disorder or other trait, an approach which has collectively characterised the functional impact of less than 1% of genetic variation. Moreover, our knowledge of variant effects is focused on the best-understood 1–2% of our DNA—the genes that encode proteins. For non-coding variation, the situation is even less certain, because the location of most known non-coding functional elements has only been recently identified [3]. Moreover, non-coding elements are not as highly conserved and their functions are often cell type and development stage specific [4].

Our lack of information about the effect of variation found through genetic testing or genome sequencing is the major barrier to the use of sequence information for diagnosing genetic disease. This lack of information limits the effectiveness of genetic precision medicine and hinders our ability to understand genome function. Even when a variant in a well-annotated functional element is known to increase disease risk, the mechanism by which it does so is often unknown. A solution lies in our ability to assess the functional effect of variants using in vitro or cell-based assays, which can provide strong evidence to interpret their biological and clinical impact and can, in principle, be applied to any variant. However, owing to the resource- and time-intensive nature of traditional variant effect assays, they have generally been undertaken reactively for individual variants only after and, in most cases long after, the first observation of the variant. Now, multiplexed assays of variant effect (MAVEs) enable the generation of ‘variant effect maps’ characterising aspects of the function of every possible single nucleotide change in a gene or functional element of interest. Because variant effect maps are comprehensive, they profile all previously observed variants, as well as those that might be found in the future. Generating variant effect maps for every protein encoding gene and regulatory element in the human genome would create an ‘Atlas’ of variant effect maps that would transform our understanding of genetics by ushering in a new era of nucleotide-resolution functional knowledge of the genome.

**Fig. 1:**
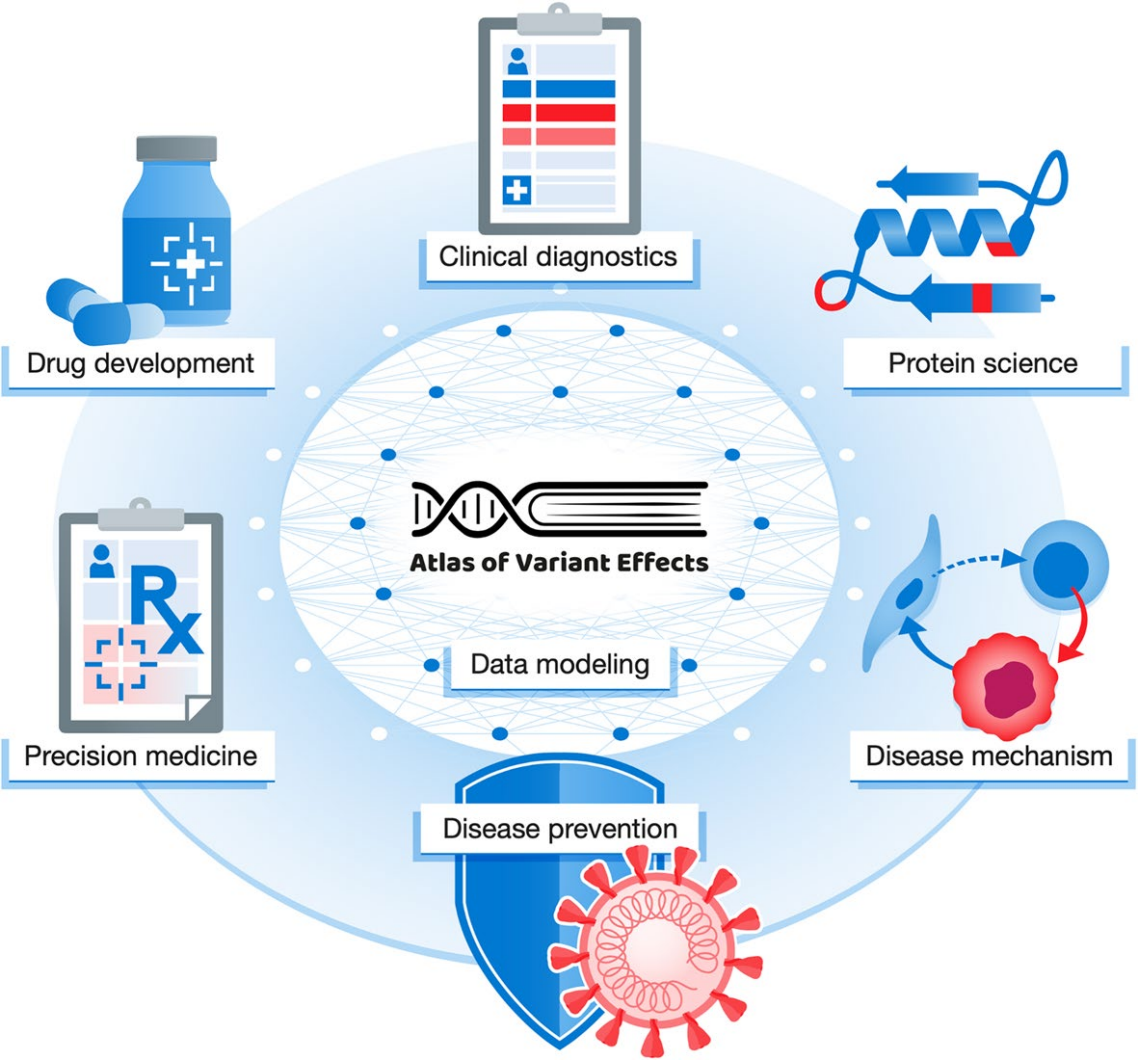
Schematic representation of areas of high impact resulting from an Atlas of Variant Effects

The generation of an Atlas of Variant Effects (AVE) would have major impact across multiple areas of basic and translational research and, importantly, for clinical care. Any effort to determine whether a variant alters function would be transformed by having an Atlas, including in the following high impact areas (Fig. 1):

- **Precision genomic medicine**. Variant effect maps of functional elements known to harbour disease-causing variation can drive more accurate, rapid and inexpensive genetic diagnostic testing. Variant effect maps can also enhance our understanding of penetrance and variable expressivity and potentially even reveal compensatory genetic perturbations. For a wide variety of genetically driven disorders, knowledge of disease risk variants allows screening within families or even populations for early detection and thus early intervention [5].
- **Disease association studies**. Just as targeted variant functional assays have assisted discovery and validation of associations between specific rare genetic variants and disease risk, variant effect maps can enable this approach broadly, at scale [6, 7].
- **Therapeutic development and pharmacogenetics**. Variant effect maps can shed light on disease mechanisms and may identify novel potential targets for drugs or other therapeutics [8], help predict the safety and efficacy of modulating specific targets, reveal routes of resistance and identify patients likely to respond favourably in clinical trials. Variant effect maps of pharmacogenes, where genetic variation can influence the activity or metabolism of drugs, could reveal the optimal dose for an individual or identify predispositions to adverse reactions. Variant effect maps could also enable the systematic study of genetic dose–response curves through functional and clinical correlations.
- **Sequence/structure/function relationships**. Understanding the relationship between sequence and function is fundamental to biology [9] and remains difficult to predict. Variant effect maps can illuminate this relationship, for example by improving or benchmarking computational variant effect prediction; revealing protein function, allostery or structure; and discerning the composition and mechanisms of regulatory elements [10–19].
- **Evolutionary genetics**. Differences in the biology of species, including those of commercial interest, is genomically encoded. Variant effect maps can highlight the subset of genetic differences between species that have functional consequences, probe inferred ancestral sequences [20] and improve phylogenetic inference [21–23].
- **Pathogen biology**. Genetic variation in pathogen genomes influences key characteristics of pathogen biology, including virulence, transmission, immune evasion and drug resistance. Variant effect maps can inform the surveillance of pathogen evolution [24] and provide opportunities to respond more rapidly, as well as revealing drug resistance and immune evasion variants [25].

By comprehensively capturing the impact of variants in functional elements throughout the genome, an Atlas of Variant Effects would accelerate and empower biological research, drug discovery and clinical practice. Systematic variant analysis, unbiased by allele frequency in any population, would empower equitable interpretation and reduce healthcare disparities [26]. Building and implementing a coherent Atlas of Variant Effects will necessarily be a collective endeavour, drawing together diverse expertise from different communities, including patients, patient advocates, researchers, clinicians, diagnostics companies and drug developers.

## MAVEs can measure the effect of genetic variants at the scale necessary to compile an Atlas of Variant Effects

**Fig. 2:**
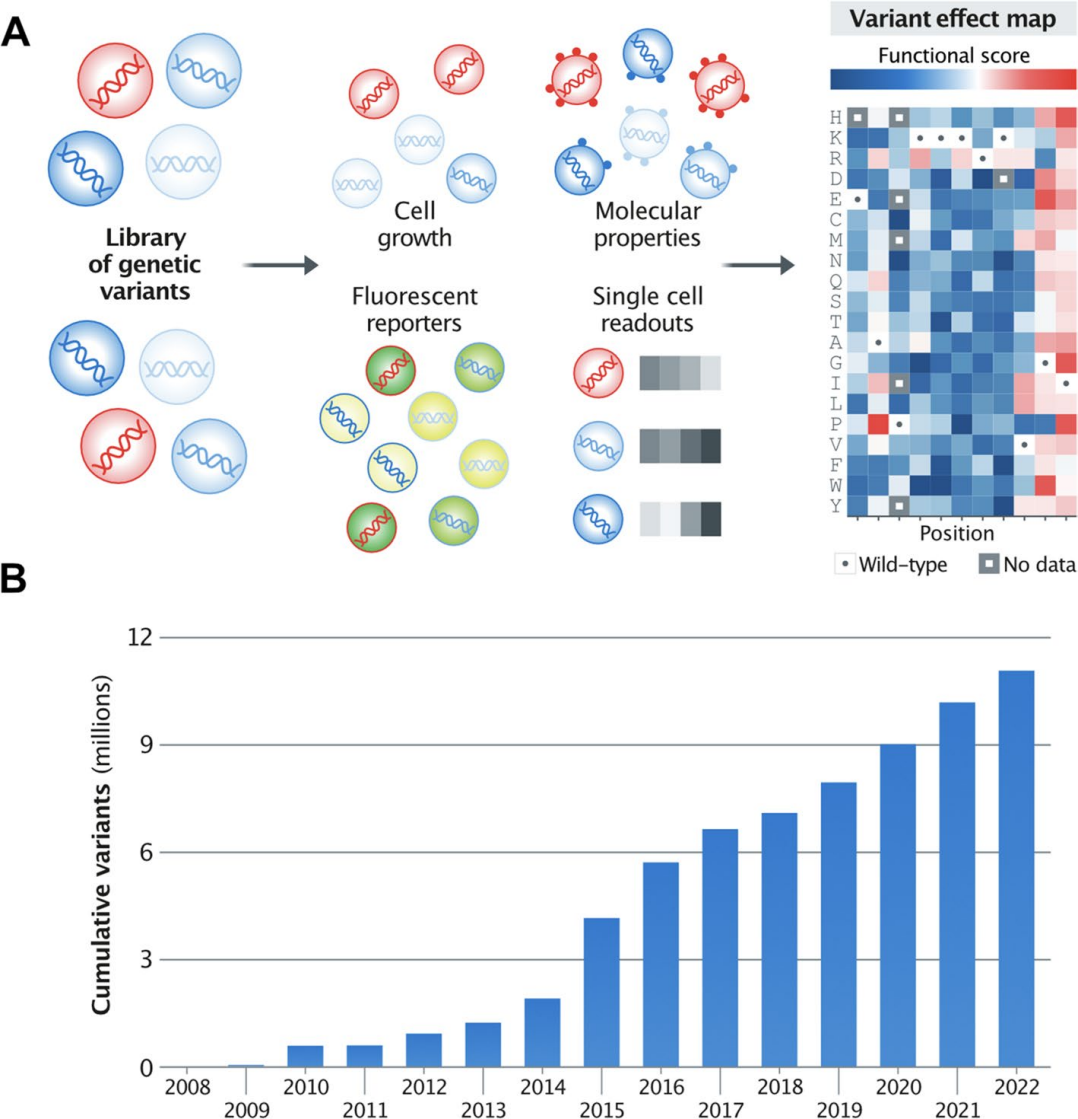
**A** (top panel) MAVEs can measure a wide variety of protein and regulatory DNA functions, and they produce comprehensive variant effect maps representing the effects of nearly all possible nucleotide or amino acid variants in the scanned functional element. A variant effect map is shown for a small region of a protein-coding gene; each column in the map is a position in a gene and each row is an amino acid substitution. Tiles are coloured based on the measured effect of the variant. **B** (bottom panel) MAVEs have been applied to hundreds of functional elements and, collectively, ~ 11 million variant effect measurements have been made with MAVEs. Data available at 10.5281/zenodo.7662580

MAVEs are a rapidly growing family of methods that involve mutagenesis of a DNA-encoded protein or regulatory element followed by a multiplexed assay for some aspect of function [9, 27–29]. High-throughput DNA sequencing is used to read out each variant’s effect in the assay (Fig. 2A). MAVEs encompass both assays of protein function, often called deep mutational scans, and of regulatory elements, often called massively parallel reporter assays. Early MAVEs were applied to small protein domains and short regulatory elements [14, 15, 30] generally querying single ‘sub-functions’ of an element such as promoter activity [14, 15], protein–ligand interactions [30–32] or stability [33, 34]. Other early efforts focused on the ability of an element to perform its overall cellular function in a cell-based growth assay [35]. Subsequently, MAVEs have been developed for a variety of functions and have been used to generate multiple variant effect maps examining different functions for the same element [9, 28, 36]. Now, MAVEs have been scaled up and optimised to enable routine application to entire genes, measuring the relative functional impact of tens of thousands of variants in a single controlled experiment.

To date, variant effect maps have been generated for hundreds of functional elements encompassing over 11 million total variants (Fig. 2B). However, existing variant effect maps cover < 1% of the known clinically relevant human genome and are largely focused on single nucleotide variants, as these are the type of variants most often encountered in current human genome sequencing and clinical testing. No functional element has been mapped in a diverse panel of cell types or across developmental stages. However, even at this very early stage in the development of a comprehensive Atlas of Variant Effects, multiplexed variant functional data are proving to be powerful. In particular, variant effect maps are beginning to reshape how human variants found in clinical genetic testing are interpreted and also to redefine our understanding of the mapping between DNA sequence and molecular, cellular and organismal phenotype.

The value of functional evidence for informing clinical variant interpretation is already well appreciated and has been incorporated within current professional guidelines for genetic diagnosis that are used internationally [37, 38]. MAVE-derived variant functional data has numerous advantages as compared to functional data derived from traditional, low-throughput assays. Unlike testing variants in small batches using different methods in different labs, MAVEs can determine the effects of thousands of variants simultaneously, not only improving reproducibility but allowing assessment of variants in the context of the functional effects of all of the variants in that gene, including the effects of known pathogenic and benign variants. Thus, MAVE-derived functional data can be used to eliminate many, if not most, of the uncertain, clinically observed variants in monogenic disease genes demonstrating the power of functional data to help deliver more definitive genetic test results to patients and clinicians [39–41].

Multiplexed variant functional data can also transform our understanding of how variants encode molecular and cellular function and how sequence dictates biological structure. For example, multiplexed measurements of variant abundance and ligand binding in SH3 and PDZ domains, combined with a model, enabled a comprehensive accounting of allostery within each domain [16]. Multiplexed variant functional data can be used to validate proposed protein structures [17, 42] or, where variant combinations are assayed, even infer them de novo [12, 13]. Knowledge of the precise mechanism of variant effects opens the door for variant-guided therapies designed to ameliorate protein misfolding or aggregation, aberrant splicing and more.

Existing variant effect maps for human genes have been generated by a range of different technologies, from yeast complementation assays to CRISPR-based saturation genome editing in human cells. Each technology has specific advantages and disadvantages. For example, yeast complementation assays are only applicable to a minority of human genes [43] and would not be appropriate for identifying some variant effects, such as those that affect functions beyond those needed for complementation or those that disrupt splicing. CRISPR-based saturation genome editing of an endogenous locus is costly and practical only for growth-based assays. Thus, no single technology can currently be used to generate maps of variant effects for all functional elements. Indeed, even within a single gene, multiple assays may be required to assess different pathophysiological mechanisms. Current MAVEs require appreciable effort, and the time and cost needed to develop new assays can be considerable. Moreover, some variant effects may only be well-modelled in terminally differentiated cell types or in multicellular systems or by assaying variant effects on complex phenotypes like cell morphology or transcriptional state. Thus, the existing portfolio of MAVE technologies can be applied to a substantial fraction of the genome, but more technology development is required to achieve comprehensive coverage of genomic functional elements and to identify the mechanism by which most variants act.

## The AVE Alliance provides international coordination to create, disseminate and implement an Atlas of Variant Effects

Compiling a complete Atlas of Variant Effects for all 20,000 human genes, not to mention potentially hundreds of thousands of noncoding regulatory elements, will require an international collaborative effort involving thousands of researchers, clinicians and technologists. Comparing this initiative to some of the landmark genomic collaborative achievements of the past 30 years highlights some of the key challenges to be addressed. The Human Genome Project (HGP) required a small number of centres generating data at unprecedented scales, in a highly coordinated and centralised fashion. By contrast, the Protein Data Bank (PDB) contains structures for thousands of human proteins, generated by thousands of researchers, in a largely uncoordinated and decentralised fashion [44]. Despite their differences, both HGP and PDB succeeded in generating an enduring and sustainable knowledge base and depended, crucially, on robust data standards, community-agreed quality metrics and centralised data deposition and dissemination. Moreover, a strong community ethos was essential for the development and adoption of these core standards and infrastructure. Some of the critical informatics infrastructure needed to support the AVE has already been developed, for example the MaveDB repository [45, 46], initial standards [47] for MAVE datasets and a MAVE project registry [48].

**Fig. 3:**
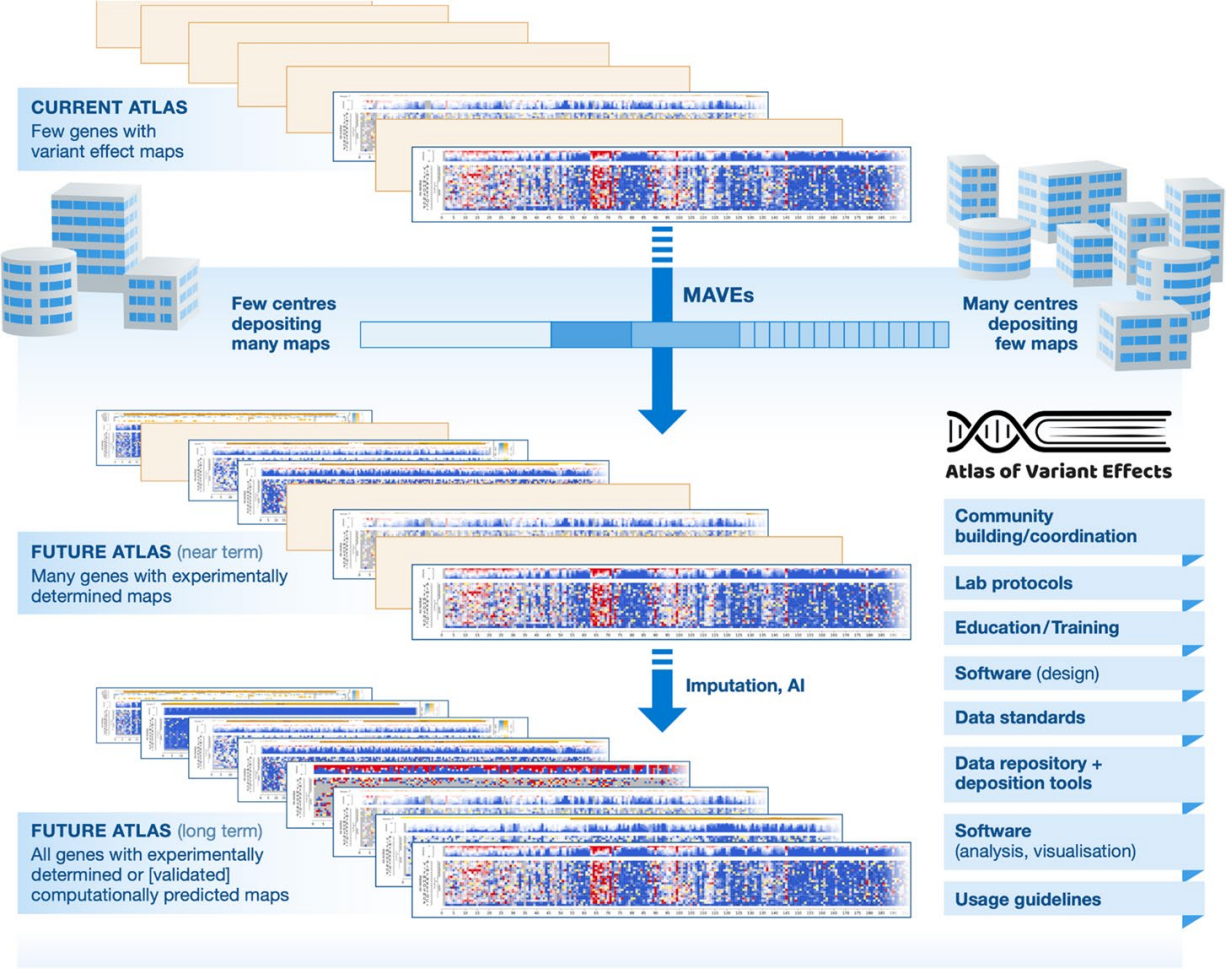
Stages of Atlas of Variant Effects completion

We envisage that the AVE will sit between the extremes exemplified by HGP and PDB, with a combination of a small number of centres generating variant effect maps at scale using generalisable assays and a large number of laboratories generating small numbers of maps, using bespoke assays, leveraging their expertise in investigating particular genes and biological pathways. Integration of variant effect data for the same gene, generated using different MAVEs, will in some cases be required to achieve accurate and comprehensive characterisation of different functional effects [39, 49–52]. The computational prediction of variant effect maps using AI/ML methods will continue to improve and will leverage growing numbers of experimentally determined variant effect maps, analogous to the advances in computational prediction of protein structures based on thousands of experimentally determined protein structures (Fig. 3). With these expectations in mind, we can identify some of the key challenges that realising the AVE vision will face and some of the likely solutions on the critical path to success:

- **Diverse expertise**. Developing new experimental technologies that reflect the complexity of biology and disease, scaling existing technologies, processing and managing complex data, and translating knowledge into clinical benefits requires a broad range of expertise, interests and competencies, working collaboratively. No one centre or community will be able to create the AVE in isolation. Technology developers, geneticists, cell biologists, protein scientists, data scientists, software engineers, clinicians will need to work together, aligned around a common vision, language and values.
- **Technology development and scaling**. Generating variant effect maps for all 20,000 genes will require both the scaling of existing technologies that can be applied to many genes, and the development of new technologies that will extend coverage of MAVE-compatible assays to all functional elements. Moreover, new approaches will be needed to assess variant effects in more complex contexts, such as specific cell types or in development, and for more complex phenotypes, such as cell morphology and behaviour.
- **Democratisation of technology**. Completing the AVE will require a major expansion in the numbers of researchers and organisations actively performing MAVEs. Readily accessible training materials, protocols, experimental resources (e.g. cell lines, libraries) and easy-to-use and flexible software will all be crucial, as will advocacy and support to facilitate researchers with expertise in informative assays to adopt MAVE technologies.
- **Data standards and coordination**. Data standards, community-agreed quality standards, centralised data deposition, open dissemination and a FAIR ethos [53] are all necessary but not sufficient for compiling the Atlas of Variant Effects. The existing informatics infrastructure needs to evolve, become integrated into the wider clinical and biological data ecosystem and be actively sustained for long term impact. Moreover, community-wide adoption of best practices with regard to data and meta-data deposition are critical for data integration.
- **Ensuring trustworthy clinical adoption**. The potential clinical impact of the Atlas of Variant Effects can only be achieved through rigorous and clinician-trusted integration into diagnostic workflows. Co-development of quality standards and guidelines with clinical communities will help to build trust, as will starting conservatively. Integration with existing clinical decision support software (e.g. DECIPHER [54]) and data resources (e.g. ClinVar [55]), as opposed to requiring diagnosticians to use new systems, will facilitate rapid adoption.

To achieve the AVE vision and tackle these challenges, an international group of diverse researchers, clinicians and diagnosticians established the Atlas of Variant Effects Alliance (www.varianteffect.org). The AVE Alliance currently has over 400 members from over 100 institutions, located in 30 countries, united by the mission to bring the AVE into reality. The AVE Alliance is committed to Open Science and places diversity and inclusion at the heart of its activities. The AVE Alliance organises an annual meeting, the Mutational Scanning Symposium, and a monthly seminar series, the Variant Effect Seminar Series. To tackle the challenges identified above, AVE has established workstreams to:

- Develop, standardise and democratise experimental and computational technologies,
- Develop the infrastructure necessary to ingest, store and disseminate high quality FAIR data,
- Ensure that clinical benefits are realised,
- Expand, coordinate and sustain a diverse and motivated community.

The AVE Alliance provides a ‘front door’ for other organisations and initiatives to work with the diverse AVE community, from complementary large-scale national initiatives such as the NIH-funded Impact of Genomic Variants on Function (IGVF), as well as research funders and commercial organisations who are keen to engage with the community as a whole. We welcome any and all readers who are interested in building and learning from the Atlas of Variant Effects to join the Alliance and get involved [56, 57].

## Data Availability

Not applicable.
